# Are minor alleles more likely to be risk alleles?

**DOI:** 10.1186/s12920-018-0322-5

**Published:** 2018-01-19

**Authors:** Takashi Kido, Weronika Sikora-Wohlfeld, Minae Kawashima, Shinichi Kikuchi, Naoyuki Kamatani, Anil Patwardhan, Richard Chen, Marina Sirota, Keiichi Kodama, Dexter Hadley, Atul J. Butte

**Affiliations:** 1 Rikengenesis Co., Ltd., 1-2-2 Ohsaki, Shinagawa-ku, Tokyo, 141-0032 Japan; 2 Preferred Networks, Inc., Otemachi Bldg. 2F, Chiyoda-ku, Tokyo, 100-0004 Japan; 3 Division of Systems Medicine, Department of Pediatrics, Stanford University, Stanford, CA 94305-5208 USA; 4 Department of Human Genetics, Graduate School of Medicine, The University of Tokyo, Bunkyo-ku, Hongo, 7-3-1, Tokyo, 113-0033 Japan; 5 AI System Department, DeNA, Inc., Shibuya 2-21-1, Tokyo, 150-8510 Japan; 6 StaGen Inc., 4-11-6, Kuramae, Taito-ku, Tokyo, 111-0051 Japan; 7 Personalis, Inc., 1330 O’Brien Dr, Menlo Park, CA 94025 USA; 8 Institute for Computational Health Sciences, University of California, 550 16th Street, San Francisco, CA 94158 USA; 9 Department of Pediatrics, University of California, San Francisco, CA USA

**Keywords:** GWASs, Complex diseases, Minor alleles, Risk alleles, Negative natural selection

## Abstract

**Background:**

Genome-wide association studies (GWASs) have revealed relationships between over 57,000 genetic variants and diseases. However, unlike Mendelian diseases, complex diseases arise from the interplay of multiple genetic and environmental factors. Natural selection has led to a high tendency of risk alleles to be enriched in minor alleles in Mendelian diseases. Therefore, an allele that was previously advantageous or neutral may later become harmful, making it a risk allele.

**Methods:**

Using data in the NHGRI-EBI Catalog and the VARIMED database, we investigated whether (1) GWASs more easily detect risk alleles and (2) facilitate evolutionary insights by comparing risk allele frequencies of different diseases. We conducted computer simulations of *P*-values for association tests when major and minor alleles were risk alleles. We compared the expected proportion of SNVs whose risk alleles were minor alleles with the observed proportion.

**Results:**

Our statistical results revealed that risk alleles were enriched in minor alleles, especially for variants with low minor allele frequencies (MAFs < 0.1). Our computer simulations revealed that > 50% risk alleles were minor alleles because of the larger difference in the power of GWASs to differentiate between minor and major alleles, especially with low MAFs or when the number of controls exceeds the number of cases. However, the observed ratios between minor and major alleles in low MAFs (< 0.1) were much larger than the expected ratios of GWAS’s power imbalance, especially for diseases whose average risk allele frequencies were low, such as myopia, sudden cardiac arrest, and systemic lupus erythematosus.

**Conclusions:**

Minor alleles are more likely to be risk alleles in the published GWASs on complex diseases. One reason is that minor alleles are more easily detected as risk alleles in GWASs. Even when correcting for the GWAS’s power imbalance, minor alleles are more likely to be risk alleles, especially in some diseases whose average risk allele frequencies are low. These analyses serve as a starting point for future studies on quantifying the degree of negative natural selection in various complex diseases.

**Electronic supplementary material:**

The online version of this article (10.1186/s12920-018-0322-5) contains supplementary material, which is available to authorized users.

## Background

Advances in genomic technologies such as DNA sequencing and single nucleotide variant (SNV) genotyping have greatly contributed to our understanding of gene-disease associations. Indeed, sophisticated genetic methods such as linkage analysis and genome-wide association studies (GWASs) have facilitated the identification of over 57,000 phenotype–genotype associations in the NHGRI-EBI Catalog (http://www.ebi.ac.uk/gwas, accessed on September 12, 2017) [1]. VARIMED [2] has also been built as a master database of disease-associated SNPs. Although GWASs have greatly improved our understanding of the genetic basis of disease risk, the missing heritability for common complex diseases remains mystifying [3, 4]. Gorlov et al. [5] reported that the overall proportion of risk alleles was higher among alleles with a frequency of < 50% (minor alleles) than among major alleles in the NHGRI-EBI Catalog. By introducing an environmental/lifestyle index to assess the influence of environmental/lifestyle factors on disease etiology, they tested the hypothesis that negative selection has different effects on environmental/lifestyle-dependent diseases than on environmental/lifestyle-independent diseases. They hypothesized that previously selectively neutral variants become risk alleles when the environment changes. Chan et al. [6] also investigated the ratio of detected risk to protective variants (R/P ratio) for several common diseases and showed that an increase in this ratio can be a strong signal of polygenic inheritance in some complex diseases (such as schizophrenia and type 2 diabetes). They found that an increase in the R/P ratio could occur if (1) there is a higher power to detect risk variants than to detect protective variants, or if (2) risk variants are present and maintained at lower frequencies because of negative selection. In addition, Park et al. [7] reported that minor alleles more often confer risk than protection, and that an inverse relationship exists between regression effects and allele frequencies.

Unlike Mendelian diseases, complex diseases are affected by both genetic and environmental factors [4, 8, 9]. Since some carriers of the risk allele responsible for a Mendelian disease have lower-than-average fitness, the frequency of the allele is uniformly low [10]. For a non-Mendelian disease, however, not only the minor allele but also the major allele of an associated locus can be the risk allele [11–14]. This may be explained by genetic drift, through which a slightly deleterious allele may have the chance to expand and become a major allele [15]. Alternatively, a neutral or advantageous allele that was previously common may become associated with a disease owing to changes in the environment, and may then serve as a risk allele for a non-Mendelian disease. In addition, overdominance, frequency-dependent selection, and gene–gene or gene–environment interactions may affect the disease allele [16].

We therefore sought to determine whether reported risk alleles for common diseases tend to be minor alleles, and if so, whether there are any biases that lead to more frequent identification of minor alleles as risk alleles. In this study, we empirically show that the reported risk alleles for non-Mendelian diseases are indeed enriched in minor alleles (with frequencies of < 0.5), particularly for SNVs with low minor allele frequencies (MAFs < 0.1). We also found that even at the same effect size, the minor allele is more likely to be identified as an associated allele, because *P*-values are lower in association studies when the minor rather than the major allele is the risk allele. Furthermore, we found that diseases with different average risk frequencies exhibit different disease characteristics (e.g., ancient/early-onset diseases versus modern/late-onset diseases), suggesting the possibility that previously selectively neutral variants become risk alleles when the environment changes. We provide supportive results for the hypothesis postulated by Gorlov et al. [5] that negative selection may have different effects on different diseases.

## Methods

### SNV–disease associations from GWAS catalog data

We downloaded the NHGRI-EBI Catalog of 57,181 associations. We filtered the data to include only those associations with *P*-values less than 5.0 × 10^− 8^ and valid risk allele frequency values (e.g., no NR (denoting that gene location information was not reported), pending, etc.). Following this, 22,566 associations for 1071 diseases and traits remained. After checking the “Initial Sample Size” column, we defined 16,084 associations as “European” and 6482 as “other.” On the basis of this classification, we collected 16,224 unique associations for 795 diseases and traits in the European population. Finally, we decided to focus our analysis on a set of 3284 SNVs for 280 diseases obtained after filtering out non-disease traits.

### SNV–disease associations from VARIMED

SNV–disease associations were obtained from VARiants Informing MEDicine (VARIMED) [2], a curated database of human SNV–disease associations. VARIMED contains 465,246 unique SNVs that can be matched with dbSNP138 SNVs. First, we selected only the associations with disease phenotypes (as opposed to non-disease traits). The selected set of associations comprised 351,162 unique SNVs. Next, we selected only the associations for which the *P*-value was < 5.0 × 10^− 8^, which reduced the size of the set to 11,957 unique SNVs. Furthermore, we focused only on associations with reported risk alleles, which were available for 7610 unique SNVs. We then focused on SNV–disease associations reported in Caucasians, which further reduced the size of unique SNVs to 6478. Finally, we mapped SNVs in VARIMED to SNVs in the 1000 Genomes Project using their genomic positions, and the resulting final set comprised 16,415 associations, including 6378 unique SNVs and 213 unique diseases.

### Disease-associated LD blocks

For each SNV–disease pair, we first attempted to identify the risk allele from the previously filtered VARIMED table. We found that of the 7556 pairs, single risk alleles were identified in 7218 pairs, whereas multiple risk alleles were identified in the remaining 338 pairs. We then filtered out the ambiguous associations and obtained a set of associations with 14,271 alleles that comprised 6179 unique SNVs, 210 unique diseases, and 7218 unique SNV–disease pairs. For each disease, we grouped SNVs with high linkage disequilibrium (LD; pairwise r^2^ > 0.8 in the European population in the 1000 Genomes Project Phase 1) into LD blocks. From each LD block, we selected the SNV with the lowest *P*-value. The risk allele frequency of the selected SNV was used as the representative allele frequency of the LD block. By extracting SNVs present in VARIMED for which we had LD information, we selected 1944 LD block SNVs for 280 diseases in European association studies in the NHGRI-EBI catalog.

### Simulation of *P*-values for association tests when major and minor alleles are risk alleles

An SNV with a low MAF is likely to have a lower chance of being discovered than a more common SNV. Even when discovered, the former has a lower chance to be included in SNV platforms than the latter. Therefore, we tested whether a minor allele was more likely to be a risk allele than a major allele using data from SNVs with similar MAFs. We first divided the SNVs into five equal MAF intervals, i.e., (0–0.1), (0.1–0.2), (0.2–0.3), (0.3–0.4), and (0.4–0.5). In each category, we performed binomial tests to examine whether the proportion of SNVs whose risk alleles were minor alleles was 0.5. Because there were five categories, the significance level was set at 0.05/5 = 0.01 according to a Bonferroni correction for multiple comparisons.

We then examined whether an SNV was more frequently identified as an associated locus when the minor allele rather than the major allele was a risk allele. Because the probability of significance in an association test is affected by sample size, effect size (such as odds ratio), penetrance, and MAF, we performed simulations under various conditions by altering the values of these parameters. In these simulations, we estimated *P*-values for the two different conditions, i.e., the minor allele as a risk allele and the major allele as a risk allele.

First, relative proportions of the three genotypes in each case and control subpopulation were calculated according to the genotype frequencies in the population (assuming Hardy–Weinberg equilibrium) and penetrance of the three genotypes (calculated from the penetrance of the lowest-risk genotype and the odds ratio, assuming an additive model). Let *p* be the minor allele frequency for a locus, and let xx, xX, and XX be the three genotypes at this locus, where x is the minor allele. Let d_1_, d_2_, and d_3_ be the penetrance for xx, xX, and XX, respectively. If the Hardy–Weinberg equilibrium holds, the frequencies of xx, xX, and XX in the population will be *p*^2^, 2*p*(1 − *p*), and (1 − *p*)^2^, respectively. The proportions of cases in the population with genotypes xx, xX, and XX will be d_1_*p*^2^, 2d_2_p(1 − *p*), and d_3_(1 − *p*)^2^, respectively, and the proportions of controls in the population with the genotypes xx, xX, and XX will be (1 − d_1_)*p*^2^, 2(1 − d_2_)*p*(1 − *p*), and (1 − d_3_)(1 − *p*)^2^, respectively. Using these proportions, the relative proportion of each genotype in the test cases will be:

$$ \mathrm{D}\left(\mathrm{xx}\right)=\frac{{\mathrm{d}}_1{\mathrm{p}}^2}{{\mathrm{d}}_1{\mathrm{p}}^2+2{\mathrm{d}}_2\mathrm{p}\left(1-\mathrm{p}\right)+{\mathrm{d}}_3{\left(1-\mathrm{p}\right)}^2} $$

$$ \mathrm{D}\left(\mathrm{xX}\right)=\frac{2{\mathrm{d}}_2\mathrm{p}\left(1-\mathrm{p}\right)}{{\mathrm{d}}_1{\mathrm{p}}^2+2{\mathrm{d}}_2\mathrm{p}\left(1-\mathrm{p}\right)+{\mathrm{d}}_3{\left(1-\mathrm{p}\right)}^2} $$

$$ \mathrm{D}\left(\mathrm{XX}\right)=\frac{{\mathrm{d}}_3{\left(1-\mathrm{p}\right)}^2}{{\mathrm{d}}_1{\mathrm{p}}^2+2{\mathrm{d}}_2\mathrm{p}\left(1-\mathrm{p}\right)+{\mathrm{d}}_3{\left(1-\mathrm{p}\right)}^2} $$

The relative proportions of the genotypes in the controls will be:

$$ \mathrm{N}\left(\mathrm{xx}\right)=\frac{\left(1-{\mathrm{d}}_1\right){\mathrm{p}}^2}{\ \left(1-{\mathrm{d}}_1\right){\mathrm{p}}^2+2\left(1-{\mathrm{d}}_2\right)\mathrm{p}\left(1-\mathrm{p}\right)+\left(1-{\mathrm{d}}_3\right){\left(1-\mathrm{p}\right)}^2} $$

$$ \mathrm{N}\left(\mathrm{xX}\right)=\frac{2\left(1-{\mathrm{d}}_2\right)\mathrm{p}\left(1-\mathrm{p}\right)}{\left(1-{\mathrm{d}}_1\right){\mathrm{p}}^2+2\left(1-{\mathrm{d}}_2\right)\mathrm{p}\left(1-\mathrm{p}\right)+\left(1-{\mathrm{d}}_3\right){\left(1-\mathrm{p}\right)}^2} $$

$$ \mathrm{N}\left(\mathrm{XX}\right)=\frac{\left(1-{\mathrm{d}}_3\right){\left(1-\mathrm{p}\right)}^2}{\left(1-{\mathrm{d}}_1\right){\mathrm{p}}^2+2\left(1-{\mathrm{d}}_2\right)\mathrm{p}\left(1-\mathrm{p}\right)+\left(1-{\mathrm{d}}_3\right){\left(1-\mathrm{p}\right)}^2} $$

Second, the relative proportion of a genotype in the test cases or control group was multiplied by the sample size. Let *n* denote the number of cases and let controls have the same sample size. The expected numbers of the genotypes in the disease group will be nD(xx), nD(xX), and nD(XX), and those in the control group will be nN(xx), nN(xX), and N(XX).

The expected numbers were rounded to obtain the numbers of each genotype in the disease and non-disease groups, and the data were analyzed by a logistic regression model using the R environment as follows:

$$ \log \left(\frac{{\mathrm{p}}_{\mathrm{d}}}{1-{\mathrm{p}}_{\mathrm{d}}}\right)=\beta {X}_1+\varepsilon \kern1em (1) $$

where P_d_ denotes the probability of the disease, *X*_1_ denotes the number of the risk alleles (0, 1, or 2) of the individual, β denotes the coefficient, and ε denotes the variable for residual variation.

Finally, the *P*-value for the association between the genotype and phenotype was calculated as follows:

1. N (for example, 500), d_1_ (for example 0.02), and *p* (for example, 0.2) were given.
2. Odds ratio r (for example, 1.3) was given, and d_2_ and d_3_ were calculated as follows:

$$ {\mathrm{d}}_2=\frac{{\mathrm{d}}_1\mathrm{r}}{1-{\mathrm{d}}_1+{\mathrm{rd}}_1} $$

$$ {\mathrm{d}}_3=\frac{{\mathrm{d}}_2\mathrm{r}}{1-{\mathrm{d}}_2+{\mathrm{rd}}_2} $$

The above equations were obtained by solving the following equations to derive the common odds ratio r from penetrance values.

$$ {\displaystyle \begin{array}{c}r=\frac{d_1}{1-{d}_1}/\frac{d_2}{1-{d}_2}\\ {}r=\frac{d_2}{1-{d}_2}/\frac{d_3}{1-{d}_3}\end{array}} $$

3. The numbers of genotypes in cases and controls were obtained by rounding the expected numbers, and a test to determine whether β in eq. (1) = 0 was performed to obtain the *P*-value.

### Running multiple simulations with a variety of parameters

We then examined the conditions under which minor alleles were more likely than major alleles to result in significance as risk alleles with a variety of parameters: the odds ratio (r), the genotype with the lowest risk (d_1_), and the number of controls (nc).

First, we fixed the number of cases (N_case = 1000) and the number of controls (N_controls = 1000). We changed the penetrance for the genotype with the lowest risk (d_1_) from 0.01 to 0.25 with increments of 0.01. We changed the minor allele frequency (p) from 0.05 to 0.50 with increments of 0.05 and the odds ratio (r) from 1.06 to 2.00 with increments of 0.01. For each parameter set (d_1_, p, r), we calculated the *P*-value with the procedure described in the previous section. We generated a graph of (p, log(*P*-value)) plots with d_1_ and r and compared the black line (minor allele as the risk allele) with the red line (major allele as the risk allele; Additional file 1: Figure S1).

**Fig. 1:**
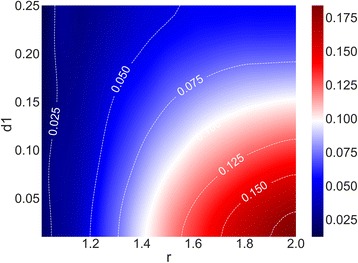
A minor allele is more likely to result in significance as a risk allele than a major allele when the odds ratio (r) is larger and the penetrance for the genotype with the lowest risk (d_1_) is smaller

We defined the relative difference (S′) between the lines as the ratio of the difference in area between the lines (S = S_black – S_red, gray color area in Additional file 1: Figure S1) to the area above the black line (total area of gray color and red color in Additional file 1: Figure S1). We plotted the relative difference in a heat map (Fig. 1) given the odds ratio (r) and the penetrance for the genotype with the lowest risk (d_1_).

Each value in the heat map (right part of the figure) shows the relative difference between the log(*P*-value) of minor and major risk allele given the odds ratio (r) and the penetrance for the genotype with the lowest risk (d_1_) in the association test simulations. The sample size was 1000 for both cases and controls. The penetrance for the genotype with the lowest risk (d_1_) ranged from 0.01 to 0.25 in increments of 0.01, and the odds ratio (r) ranged from 1.06 to 2.00 in increments of 0.01. As shown in the right part of the figure, blue in the heat map indicates that the differences are small (relative difference < 0.05), white that the differences are medium (relative difference = 0.10), and red that the differences are high (relative difference > 0.15).

Next, we fixed the total of number of cases and controls (*N* = 2000), set the penetrance for the lowest risk (d_1_ = 0.03), changed the odds ratio (r) from 1.06 to 2 with increments of 0.01, and changed the number of controls (nc) from 200 to 1800 with increments of 100. We plotted the relative difference in a heat map (Additional file 2: Figure S2) given the odds ratio (r) and the number of controls (nc).

### Statistical power calculation in NHGRI-EBI catalog studies

We reconfirmed our simulation results with studies in the NHGRI-EBI Catalog database using an online GAS power calculator, (http://csg.sph.umich.edu//abecasis/cats/gas_power_calculator/index.html). Using the same parameters (number of cases and controls) as used in the representative studies in the NHGRI-EBI Catalog, we compared the statistical powers for minor (*p* ≤ 0.5) and major risk alleles (1 − *p* > 0.5) under the following conditions: the significance level of the study design *P* < 5.0 × 10^− 8^; the disease allele frequency *p* = 0.05, 0.1, 0.15, 0.20, 0.25, 0.30, 0.35, 0.40, 0.45, and 0.50; the prevalence was selected for the target disease (for example, 0.073 for type 2 diabetes); and the disease model was multiplicative, additive, dominant, or recessive.

### Assessing the explanatory power of the observed data using GWAS power simulations

To assess the magnitude of the explanatory power of the observed data using GWAS power simulations, we compared the expected and observed proportions of SNVs whose risk alleles were minor alleles. To estimate the expected proportion, we calculated the statistical power for detecting minor alleles and that for major alleles in each MAF interval [(0–0.1), (0.1–0.2), (0.2–0.3), (0.3–0.4) and (0.4–0.5)] with parameters of real GWAS studies. For example, for a type 1 diabetes study by Barrett et al. [17], we simulated the GWAS power calculations with 7514 cases and 9054 controls (significance level = 5.0 × 10^− 8^, prevalence = 0.002, genotype relative risk = 1.15, assuming a multiplicative disease model). We calculated the statistical powers of detecting minor alleles (y_minor) and major alleles (y_major) for each interval. For example, for the (0–0.1) interval, we compared the statistical power when the risk allele frequency was 0.05 (y_0.05) and 0.95 (y_0.95) using the method described in the previous section. The expected proportion was calculated by y_minor / (y_minor + y_major). We then calculated the *P*-value by conducting the binomial test with the null hypothesis that the observed proportion was the expected proportion.

## Results

**Fig. 2:**
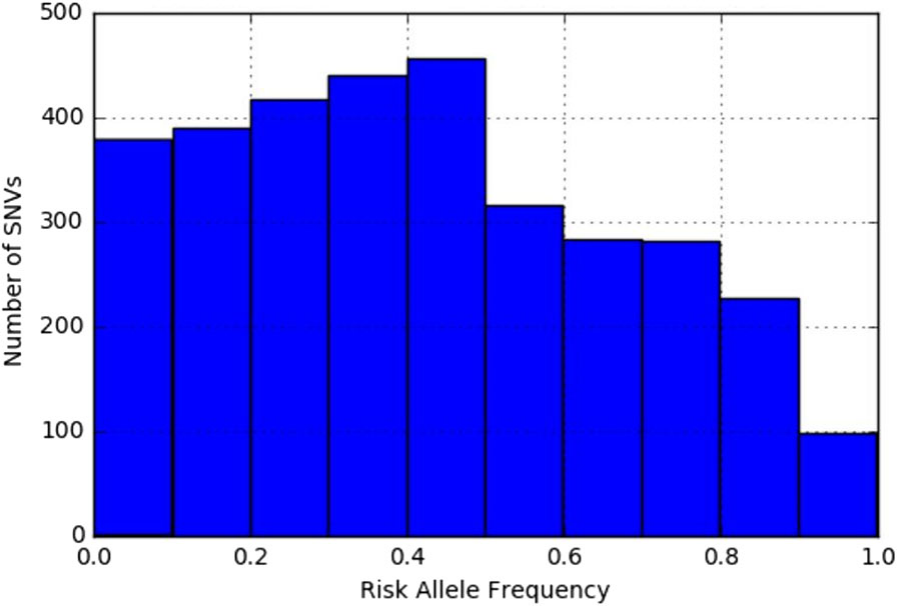
Risk allele frequencies for 3284 SNVs in 280 diseases extracted from the NHGRI-EBI Catalog of European association studies

The distribution of risk allele frequencies for 3284 SNVs in 280 diseases were extracted from the NHGRI-EBI Catalog (Fig. 2). We observed a clear enrichment of minor alleles (risk allele frequency < 0.5); 63.4% of the SNVs had risk allele frequencies of < 0.5, whereas 36.6% of SNVs had risk allele frequencies of > 0.5 (average, 0.419).

The hypothesis that a risk allele is more likely to be a minor allele was supported by a similar analysis carried out using the curated associated SNVs from VARIMED [2]. Slight enrichment of the rare SNVs (risk allele frequency < 0.5) was replicated in the VARIMED database (Additional file 3: Figure S3). The average risk allele frequency for the SNVs from VARIMED was 0.46, whereas the fraction of SNV–disease associations with a risk allele frequency < 0.5 was 57%.

The actual discovery of SNVs is largely biased by MAFs. Thus, SNVs with very low (e.g., < 0.01) MAFs have a lower chance of being discovered or associated with a disease than those with higher frequencies. In addition, SNVs with very low MAFs are less likely to be included in SNV arrays. Therefore, we classified disease-associated SNVs into five categories according to their MAFs. For each category, the number of SNVs in which minor alleles were the risk alleles was compared with the number of SNVs in which major alleles were the risk alleles. In each of the categories, the former was significantly larger (*P* < 0.01 with Bonferroni correction) than the latter (Table 1). The proportion of SNVs in which minor alleles were the risk alleles was high, particularly for SNVs with small MAFs; for example, for the interval (0–0.1), the proportion was 0.794, while the proportions were 0.591–0.631 for the other intervals (Table 1). There are two possible explanations for the above trend: (a) risk alleles are more likely to be minor alleles, or (b) association tests are more likely to result in significance when minor alleles rather than major alleles are risk alleles. The latter possibility was assessed by simulation.

**Table 1:** SNVs with different MAFs whose risk alleles are minor alleles

MAF interval	Total number	SNVs whose risk alleles are minor alleles	Proportion^a^	Lower limit^b^	Upper limit^b^	*P*-value^c^
(0–0.1)	476	378	0.794	0.755	0.830	< 2.2E-16
(0.1–0.2)	616	389	0.631	0.592	0.670	6.8E-11
(0.2–0.3)	698	417	0.597	0.560	0.634	2.97E-07
(0.3–0.4)	723	440	0.609	0.572	0.644	5.75E-09
(0.4–0.5)	771	456	0.591	0.556	0.626	4.29E-07

^a^Proportion of SNVs whose risk alleles are minor alleles

^b^Lower and upper limits of the 95% confidence interval for the proportion as determined by the Clopper–Pearson method

^c^*P*-value for the binomial test with the null hypothesis that the proportion is 0.5

**Fig. 3:**
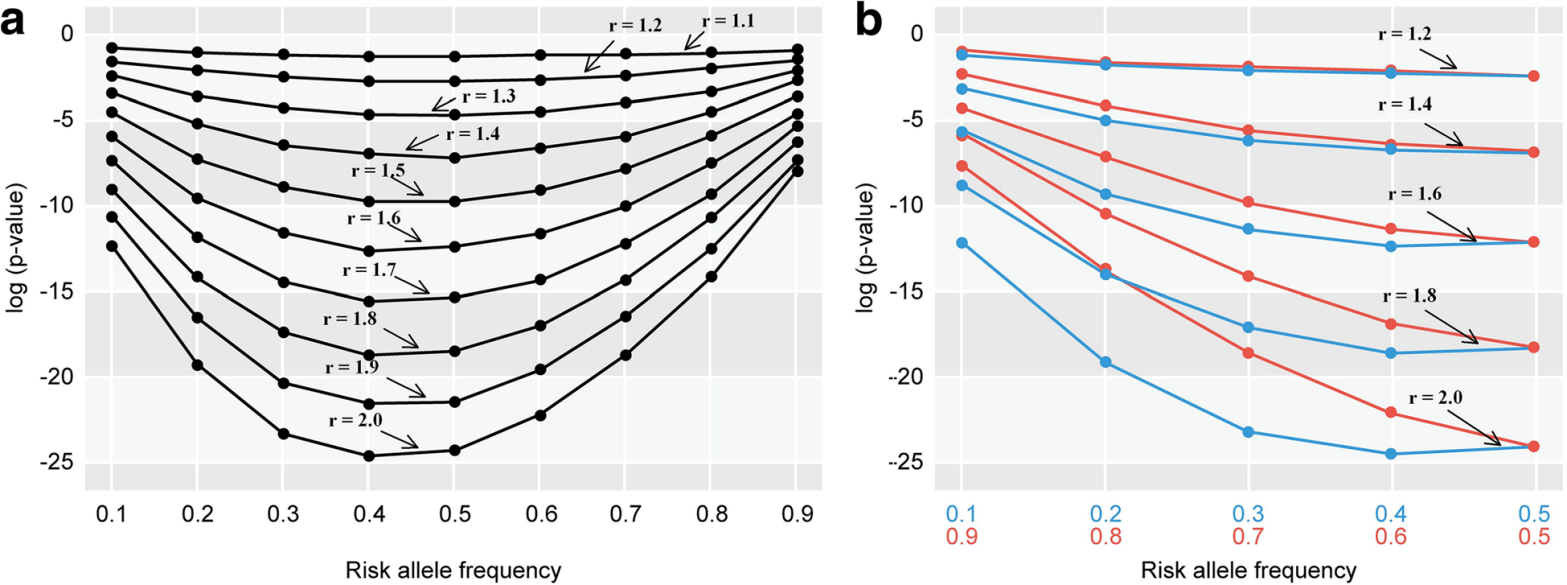
**a** Log-transformed *P*-values of association tests determined by logistic regression analysis with odds ratio increments of 0.1. The sample size was 1000 for both cases and controls. The penetrance for the genotype with the lowest risk was 0.03, and the odds ratio (r) ranged from 1.1 to 2.0 in increments of 0.1. **b** Log-transformed *P*-values of association tests determined by logistic regression analysis with odds ratio increments of 0.2. The sample size was 1000 for both cases and controls. The penetrance for the genotype with the lowest risk was 0.03, and the odds ratio (r) ranged from 1.2 to 2.0 in increments of 0.2. Red and blue circles/lines indicate values for risk allele frequencies shown in red and blue, respectively

We examined whether an SNV was more frequently identified as an associated locus when the minor allele rather than the major allele was a risk allele. Since the probability of significance in an association test is affected by sample size, effect size (such as odds ratio), penetrance, and MAF, we performed simulations under various conditions by changing the values of these parameters. In these simulations, we estimated *P*-values for the two different conditions, i.e., the minor allele as a risk allele and the major allele as a risk allele. We found that *P*-values in logistic regression analysis tended to be lower when minor alleles were risk alleles. Different sample sizes, penetrance levels for the lowest-risk genotype, and odds ratios between the lowest-risk genotype and the heterozygote were examined. Odds ratios between the heterozygote and the highest-risk genotype, as well as MAFs, were also examined (Fig. 3a, b, and Additional file 4: Figure S4). The results consistently indicated that the *P*-value of the association test was generally lower when the minor rather than the major allele was the risk allele. The differences in *P*-values of the association test between the minor (risk allele frequency: *p*) and major risk allele (risk allele frequency: 1 − *p*) progressively decreased as *p* approached 0.5 (Fig. 3b).

We then visualized the relationships between the parameters (r: odds ratio, d_1_: penetrance for the genotype with the lowest risk) and the degree of differences between the log(*P*-values) when the minor or major allele was the risk allele (Fig. 1). The differences were larger when r was larger and d_1_ was smaller, and they were relatively small when r was less than 1.2 or d_1_ was larger than 0.24. Additional file 2: Figure S2 shows heat map plots for r and the number of controls (nc) for the relative differences in log(*P*-values) between the minor and major allele as the risk allele. The differences were larger when r and nc were larger, and they were relatively small when r was less than 1.1 or nc was less than 300.

We also confirmed our results with real examples in the NHGRI-EBI Catalog using the statistical power test of an online GAS power calculator. The statistical power was indeed greater for risk alleles that were found at *p* = 0.05 (risk allele frequency, 5%) than at *p* = 0.95 (risk allele frequency, 95%), assuming multiplicative, additive, and dominant disease models. For example, one of the GWASs for late-onset Alzheimer’s disease (PubMed ID: 24,162,737) used 17,008 Caucasian patients and 37,154 Caucasian control individuals. Under the multiplicative disease model, if prevalence = 0.05, genotype relative risk = 1.15, and the significance level of the study design *P* < 5.0 × 10^− 8^, then the statistical power (y) was 0.326 when the risk allele frequency (*p*) was 0.05 (y_0.05 = 0.326), whereas y was 0.256 when *p* was 0.95 (y_0.95 = 0.256). The statistical power was also greater for the risk alleles that were found at *p* = 0.10 (y_0.10 = 0.922) than at *p* = 0.90 (y_0.90 = 0.878). The difference in statistical power between *p* = 0.10 and *p* = 0.90 (y_0.10 – y_0.90 = 0.044) was smaller than the difference in statistical power between *p* = 0.05 and *p* = 0.95 (y_0.05 − y_0.95 = 0.07). When *p* (*p* < 0.1) is larger, the difference is smaller. When *p* is larger than 0.1 (0.1 < *p* < 0.5), the statistical powers of both *p* and 1 − *p* are almost 1.0. Assuming the additive model, the result was quite similar to that of the multiplicative model. Assuming the dominant model, the difference in y between *p* = 0.05 (y_0.05 = 0.235) and *p* = 0.95 (y_0.95) = 0.0 was much larger than that in the additive model. On the other hand, assuming the recessive model, y of *p* = 0.05 (y_0.05 = 0) was smaller than that of *p* = 0.95 (y_0.95 = 0.191). The same trends have been observed in other studies. We showed some examples of statistical power analyses in real studies on type 1 diabetes, type 2 diabetes, schizophrenia, and myopia (Additional file 5: Figure S5, Additional file 6: Figure S6, Additional file 7: Figure S7, and Additional file 8: Figure S8).

To assess the magnitude of the explanatory power of the observed data using GWAS power simulations, we compared the expected proportion of SNVs whose risk alleles were minor alleles with the observed proportion. Table 2 shows the comparisons of the observed and expected proportions in myopia. The statistical power of detecting minor and major alleles was calculated by the methods explained in the previous paragraph given the parameters according to Meng et al. [18]. For example, under the multiplicative disease model, if prevalence = 0.25, genotype relative risk = 1.60, and the significance level of the study design *P* < 5.0 × 10^− 8^, then the statistical power (y) was 0.02 when the risk allele frequency (p) was 0.05 (y_0.05 = 0.02), whereas y was 0.01 when p was 0.95 (y_0.95 = 0.01). A minor allele (y_0.05 = 0.02) was more likely to result in significance as a risk allele than a major allele (y_0.95 = 0.01). The expected proportion of SNVs whose risk alleles were minor alleles was 0.67 (y_0.05 / (y_0.05 + y_0.95) = 0.67). When we consider this GWAS’s power imbalance, the *P*-value for the binomial test was significant (*P* = 0.0015), given the null hypothesis that the observed proportion (observed prop = 1.0) is the expected proportion (expected_prop = 0.67). When we do not consider the GWAS’s power imbalance, the *P*-value for the binomial test was much smaller (*P* = 0.0000076), given the null hypothesis that the observed proportion is 0.5. For each MAF interval, the number of SNVs in which minor alleles were the risk alleles was compared with the expected number of SNVs in which major alleles were the risk alleles. In the interval (0–0.1), the former was significantly larger (*P* < 0.01 with Bonferroni correction) than the latter (*P* = 0.0015; Table 2). The average of the risk allele frequency of 31 SNVs was 0.250, and 23 of the total 31 (74.2%) were minor alleles. Twenty-one of the 23 (91.3%) minor risk alleles had risk allele frequencies less than 0.2.

**Table 2:** Comparisons of observed and expected proportions of SNVs whose risk alleles were minor alleles in myopia

MAF interval	SNVs whose risk alleles were minor alleles	SNVs whose risk alleles were major alleles	Observed proportion^a^	Statistical power of detecting minor alleles	Statistical power of detecting major alleles	Expected proportion^b^	*P*-value^c^ (Original)	*P*-value^d^ (Considering the GWAS’s power imbalance)	Lower limit^e^ (Considering the GWAS’s power imbalance)	Upper limit^e^ (Considering the GWAS’s power imbalance)
(0, 0.1)	18	0	1.0	0.002	0.001	0.67	0.0000076*^f^	0.0015 *^f^	0.81	1.00
(0.1, 0.2)	3	3	0.5	0.100	0.059	0.63	1.00	0.68	0.11	0.88
(0.2, 0.3)	2	2	0.5	0.330	0.248	0.57	1.00	0.66	0.05	0.85
(0.3, 0.4)	0	2	0	0.507	0.444	0.53	0.50	0.22	0.00	0.84
(0.4, 0.5)	0	1	0	0.579	0.558	0.51	1.00	0.49	0.00	0.975

The parameters for the statistical power calculation were chosen according to Meng et al. [18]: Cases = 190, controls = 1064, significance level = 5.0E-08, prevalence = 0.25, genotype relative risk = 1.60

^a^Proportion of SNVs whose risk alleles were minor alleles

^b^Expected proportion of SNVs whose risk alleles were minor alleles (Considering the GWAS’s power imbalance)

^c^*P*-value for the binomial test with the null hypothesis that the observed proportion is 0.5

^d^*P*-value for the binomial test with the null hypothesis that the observed proportion is the expected proportion

^e^Lower and upper limits of the 95% confidence interval for the proportion by the Clopper–Pearson method with the null hypothesis that the observed proportion is the expected proportion

^f^*P-value ≤0.01

As in myopia, in sudden cardiac arrest and systemic lupus erythematosus, which have low MAFs (< 0.1 or 0.1 ≤ MAFs < 0.2), the observed excess in the ratios of minor to major alleles was much larger than the expected excess considering GWAS power imbalance (observed proportion > expected proportion) (Additional file 9: Tables S1 and S2). For example, in the interval (0.1–0.2) of sudden cardiac arrest studies (2 studies), the number of SNVs in which minor alleles were the risk alleles was significantly larger (*P* < 0.01 with Bonferroni correction) than the expected number of SNVs in which major alleles were the risk alleles (*P* = 0.0078; Additional file 9: Table S1). In the 2 studies on sudden cardiac arrest, the average risk allele frequency of 13 SNVs (12 SNVs were reported in [https://www.ncbi.nlm.nih.gov/pubmed/21658281] and 1 in [https://www.ncbi.nlm.nih.gov/pubmed/21738491]) was 0.121, and all SNVs were minor alleles. Twelve of the 13 (92.3%) minor risk alleles had risk allele frequencies less than 0.2. In the interval (0.1–0.2) of systemic lupus erythematosus studies (6 studies), the number of SNVs in which minor alleles were the risk alleles was significantly larger (*P* < 0.01 with Bonferroni correction) than the expected number of SNVs in which major alleles were the risk alleles (*P* = 0.00031; Additional file 9: Table S2). In the 6 studies on systemic lupus erythematosus, the average risk allele frequency of 32 SNVs was 0.203, and 31 of the total 32 SNVs (96.8%) were minor alleles. Twenty of the 32 (62.5%) minor risk alleles had risk allele frequencies < 0.2, and no major risk alleles had risk allele frequencies < 0.2 (100% of the 20 SNPs were minor alleles in the (0–0.2) interval).

## Discussion

Our analyses showed that minor alleles exhibit a greater tendency to be risk alleles, especially when the minor risk allele frequency is below 0.1. We investigated whether any biases exist in the identification of risk alleles, leading to more frequent identification of minor alleles as risk alleles. Our statistical simulations showed that association tests were more likely to result in significance when minor alleles rather than major alleles were risk alleles. The differences in detecting power of GWASs between minor and major alleles become, larger particularly with low minor allele frequencies and higher numbers of controls than case samples. The discrepancy between the proportion of minor and major alleles that are risk alleles increases almost linearly with risk allele frequency. Our results support a recent independent study by Chan et al. [6]; using a different simulation model, they reported more power to detect risk variants than to detect protective variants in GWAS summary association statistics. However, our statistical simulation of *P*-values could not fully explain why this tendency was greater than expected. For example, the proportion of minor alleles that were risk alleles in the MAF category of (0.4–0.5) was approximately 60%, which was higher than our expected value of around 50% when the risk allele frequency was 0.5. Furthermore, the observed excess in the ratios of minor to major alleles was much larger than expected in myopia, sudden cardiac arrest, and systemic lupus erythematosus, especially in low MAFs (< 0.1).

As suggested by Chan et al. [6], an increase in the R/P ratio can occur if risk variants are present and have been maintained at low frequencies by negative selection. We therefore considered our results from an evolutionary viewpoint.

Numerous germline mutations are removed immediately after being generated, either by selection or randomly. The retained variants are maintained and may expand in the population; however, some mutations may, over time, cause diseases and disorders owing to environmental changes. If a disease-associated allele has a lower fitness from the beginning, it is not likely to become a major allele. However, an allele that has become disease-related owing to an environmental change can be a major allele. In our study, the deviation from 0.5 in the proportion of SNVs in which minor alleles were the risk alleles was rather small (0.591–0.631) when the MAF was relatively high (> 0.1), indicating that most of the SNVs with those MAFs were associated with diseases resulting from environmental changes. Moreover, the rather small deviation from 0.5 may be explained by a preference of association studies to detect significance in SNVs in which minor alleles are the risk alleles. However, the predominance of SNVs in which minor alleles are the risk alleles (79.4%) among those with low MAFs (< 0.1) may reflect the fact that numerous risk alleles in this category might be the result of mutations that occurred recently (probably a few thousand to ten thousand years ago).

**Fig. 4:**
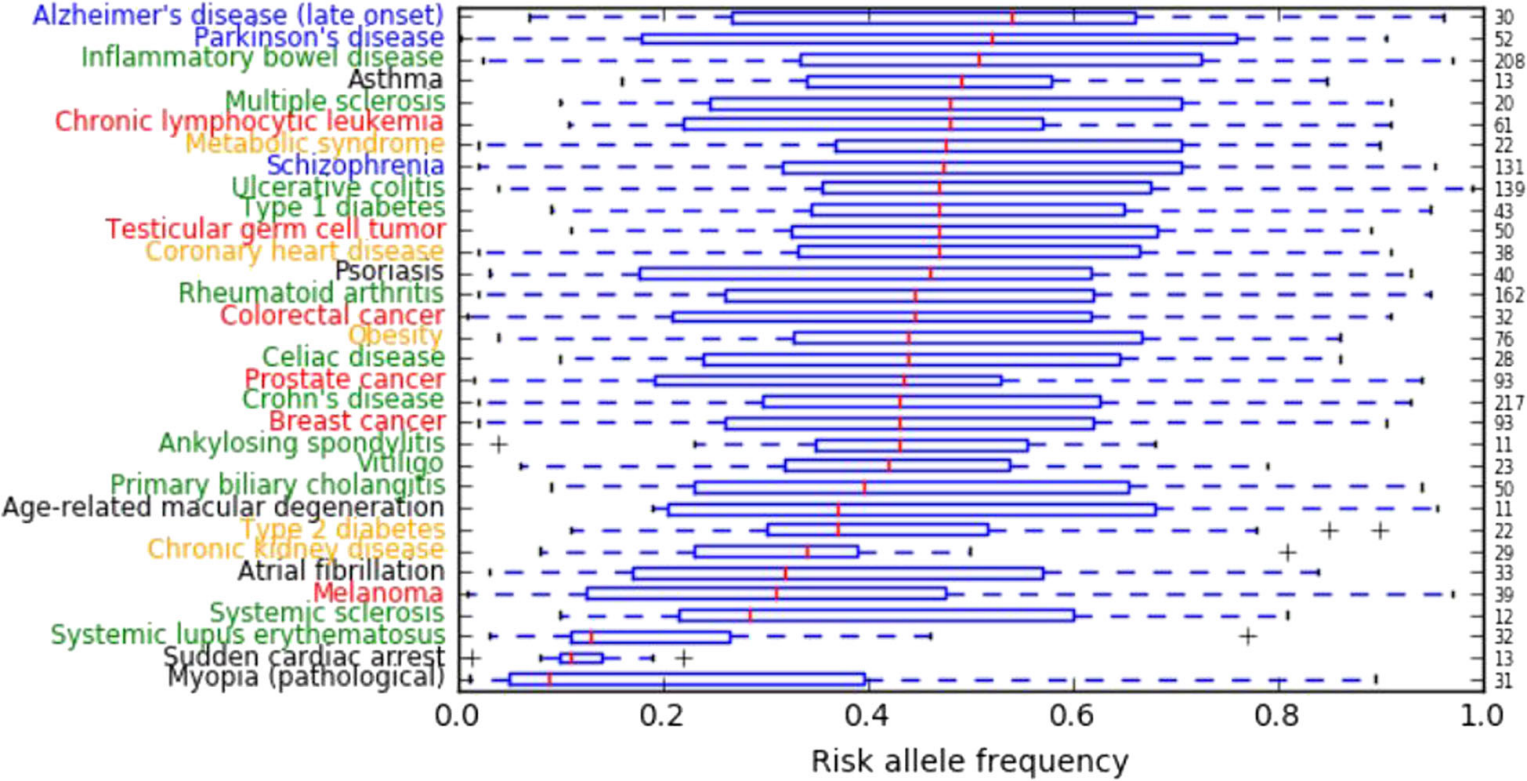
Distribution of disease-specific risk allele frequencies for major diseases in the NHGRI-EBI Catalog. Neuropsychiatric diseases (blue), autoimmune diseases (green), metabolic and cardiovascular diseases (orange), cancer (red), and unclassified diseases (black) are shown. Numbers on the right-hand side denote the number of SNVs

According to the analysis of NHGRI-EBI Catalog data, diseases whose average risk allele frequencies were low included myopia, sudden cardiac arrest, systemic lupus erythematosus, systemic sclerosis, melanoma, atrial fibrillation, and chronic kidney disease (Fig. 4). In contrast, diseases whose average risk allele frequencies were high included Alzheimer’s disease (late onset), Parkinson’s disease, inflammatory bowel disease, multiple sclerosis, chronic lymphocytic leukemia, metabolic syndrome, schizophrenia, ulcerative colitis, and type 1 diabetes (Fig. 4).

We speculate that for diseases whose average risk allele frequencies were low, negative natural selection had long been keeping deleterious mutations at a very low frequency until recently, for instance, myopia (before the advent of glasses) or sudden cardiac arrest. Chan et al. [6] suggested that an excess of risk variants compared to protective variants can be caused by negative selection. They simulated negative selection and observed an increase in the R/P ratio for the frequency bins within 1–15% but not for the 30–50% bin. They concluded that risk variants would be kept at lower frequencies, whereas protective variants would drift to higher frequencies. In Table 2, we showed that the observed excess in the ratios of minor to major alleles were much larger than the expected ratios of GWAS power imbalance in myopia. We think that natural selection has had enough time to keep the frequency of the risk allele of myopia low.

Meanwhile, for diseases whose average risk allele frequencies were high, we speculate that recent environmental changes (including epigenetic changes, microbiome changes, or other factors) might play a crucial role. For example, diabetic traits may have been beneficial in a low-energy environment in the past [19]. Modern psychological disorders may be largely influenced by the complexity of human communication in current times. Coronary heart disease may not have occurred frequently in individuals with low fat intake, characteristic of ancient human diets, and Alzheimer’s disease may not have been a major problem in the past, because average life expectancy was not very high. Our speculation is supported by the recent research of Gorlov et al. [5], who hypothesized that negative selection may have different effects on environment/lifestyle-dependent and -independent diseases. They suggested that environment/lifestyle-dependent diseases tend to have a higher frequency of risk-associated variants, suggesting a weak effect of negative selection. We think that natural selection has not had sufficient time to influence the frequencies of environment/lifestyle-dependent diseases.

There are several limitations to our approach. First, the data used for this analysis were based on manual curation from previous publications, which may have introduced errors or publication bias. Second, because most GWASs have been carried out on Caucasians, we excluded non-Caucasian studies and thus did not explore whether our findings are consistent across other populations. Third, because most of the associations that we explored here were obtained through GWASs, rare variants associated with diseases were not included. Similar analyses on rare variants should be carried out for other populations of interest using advanced genome sequencing technologies. Finally, most of the variants listed in the NHGRI-EBI Catalog are merely SNVs that tag risk, as opposed to being causal SNVs. Because of this, we need to interpret the results of Fig. 4 carefully.

## Conclusions

In summary, we reported that the risk alleles from GWASs of common diseases tend to be minor alleles in both the NHGRI-EBI Catalog and the VARIMED database. Notably, our computer simulations revealed that one reason was the larger difference in the power of GWASs to differentiate between minor alleles and major alleles, particularly for studies with low MAFs or those with more controls than case samples. However, we found that the observed excess in the ratios of minor to major alleles in low MAFs (< 0.1) were much larger than the expected ratios of GWAS power imbalance, especially for diseases whose average risk allele frequencies were low, such as myopia and sudden cardiac arrest. We speculate that this could be the result of negative natural selection; however, further systematic studies are necessary to confirm this possibility.

## Additional files

Additional file 1: Figure S1. Calculating the relative difference (S′) in (*p*, log(*P*-value)) plots. The y-axis represents the log(*P*-value), and the x-axis represents the risk allele frequency (*p*). The relative difference (S′) was defined as the ratio of the area between the black line (minor allele as the risk allele) and the red line (major allele as the risk allele) to the maximum area surrounded by the black and red lines. (TIFF 479 kb)

Additional file 2: Figure S2. A minor allele is more likely to result in significance as a risk allele than a major allele when the odds ratio (r) and the number of controls (nc) are larger. Each value in the heat map (right part of the figure) shows the relative difference between the log(*P*-value) of a minor and major risk allele given the odds ratio (r) and the number of controls (nc) in the association test simulations. The total number of cases and controls was 2000 (*n* = 2000). The penetrance for the genotype with the lowest risk (d_1_) was 0.03, and the number of controls (nc) ranged from 200 to 1800 in increments of 100. The odds ratio (r) ranged from 1.06 to 2.00 in increments of 0.01. As shown in the right part of the figure, blue in the heat map indicates that the differences are small (relative difference < 0.05), white that differences are medium (relative difference = 0.10), and red that differences are high (relative difference > 0.15). (JPEG 3725 kb)

Additional file 3: Figure S3. Risk allele frequencies of LD block SNVs for 213 diseases extracted from the VARIMED database of European association studies. (TIFF 156 kb)

Additional file 4: Figure S4. Log-transformed *P*-values of association tests determined by logistic regression analysis with penetrance for genotype with the lowest risk of 0.01 and odds ratio increments of 0.2. The sample size was 1000 for both cases and controls. The penetrance for the genotype with the lowest risk was 0.01, and the odds ratio (r) ranged from 1.1 to 2.0 in increments of 0.2. Red and black circles/lines indicate values for risk allele frequencies shown in red and black, respectively. (TIFF 570 kb)

Additional file 5: Figure S5. Statistical power analysis for type 1 diabetes study [17]. The number of cases was 7514, and the number of controls was 9045. We assumed that the significance level of the study design was 5.0 × 10^− 8^, the prevalence was 0.002, the genotype relative risk was 1.15, and the disease model was multiplicative. The graph shows the relationships between disease allele frequency (x-axis) and the statistical power (y-axis). The right top table shows the difference between the statistical power in the minor and major risk allele frequency (*p* = 0.05 vs. *p* = 0.95, *p* = 0.15 vs. *p* = 0.85, *p* = 0.25 vs. *p* = 0.75, *p* = 0.35 vs. *p* = 0.65, and *p* = 0.45 vs. *p* = 0.55). (TIFF 689 kb)

Additional file 6: Figure S6. Statistical power analysis for type 2 diabetes study [20]. The number of cases was 4595, and the number of controls was 5579. We assumed that the significance level of the study design was 5.0 × 10^− 8^, the prevalence was 0.073, the genotype relative risk was 1.2, and the disease model was multiplicative. The graph shows the relationships between disease allele frequency (x-axis) and the statistical power (y-axis). The right top table shows the difference between the statistical power in the minor and the major risk allele frequency (*p* = 0.05 vs. *p* = 0.95, *p* = 0.15 vs. *p* = 0.85, *p* = 0.25 vs. *p* = 0.75, *p* = 0.35 vs. *p* = 0.65, and *p* = 0.45 vs. *p* = 0.55). (TIFF 757 kb)

Additional file 7: Figure S7. Statistical power analysis for schizophrenia study [21]. The number of cases was 5001, and the number of controls was 6243. We assumed that the significance level of the study design was 5.0 × 10^− 8^, the prevalence was 0.01, the genotype relative risk was 1.2, and the disease model was multiplicative. The graph shows the relationships between disease allele frequency (x-axis) and the statistical power (y-axis). The right top table shows the difference between the statistical power in the minor and major risk allele frequency (*p* = 0.05 vs. *p* = 0.95, *p* = 0.15 vs. *p* = 0.85, *p* = 0.25 vs. *p* = 0.75, *p* = 0.35 vs. *p* = 0.65, and *p* = 0.45 vs. *p* = 0.55). (TIFF 777 kb)

Additional file 8: Figure S8. Statistical power analysis for myopia study [18]. The number of cases was 190, and the number of controls was 1064. We assumed that the significance level of the study design was 5.0 × 10^− 8^, the prevalence was 0.25, the genotype relative risk was 1.6, and the disease model was multiplicative. The graph shows the relationships between disease allele frequency (x-axis) and the statistical power (y-axis). The right top table shows the difference between the statistical power in the minor and major risk allele frequency (*p* = 0.05 vs. *p* = 0.95, *p* = 0.15 vs. *p* = 0.85, *p* = 0.25 vs. *p* = 0.75, *p* = 0.35 vs. *p* = 0.65, and *p* = 0.45 vs. *p* = 0.55). (TIFF 646 kb)

Additional file 9: Table S1. Comparisons of observed and expected proportions of SNVs whose risk alleles are minor alleles in sudden cardiac arrest. **Table S2.** Comparisons of observed and expected proportions of SNVs whose risk alleles are minor alleles in systemic lupus erythematosus. (DOCX 16 kb)

## Abbreviations

GWASs: Genome-wide association studies
MAFs: Minor allele frequencies
R/P: The ratio of detected risk to protective variants
SNV: Single nucleotide variant
VARIMED: VARiants Informing MEDicine
