# Supplementary figures and images for: Are minor alleles more likely to be risk alleles?
Source: BMC Med Genomics. 2018 Jan 19;11:3. doi: 10.1186/s12920-018-0322-5 (PMC5775585; doi:10.1186/s12920-018-0322-5)

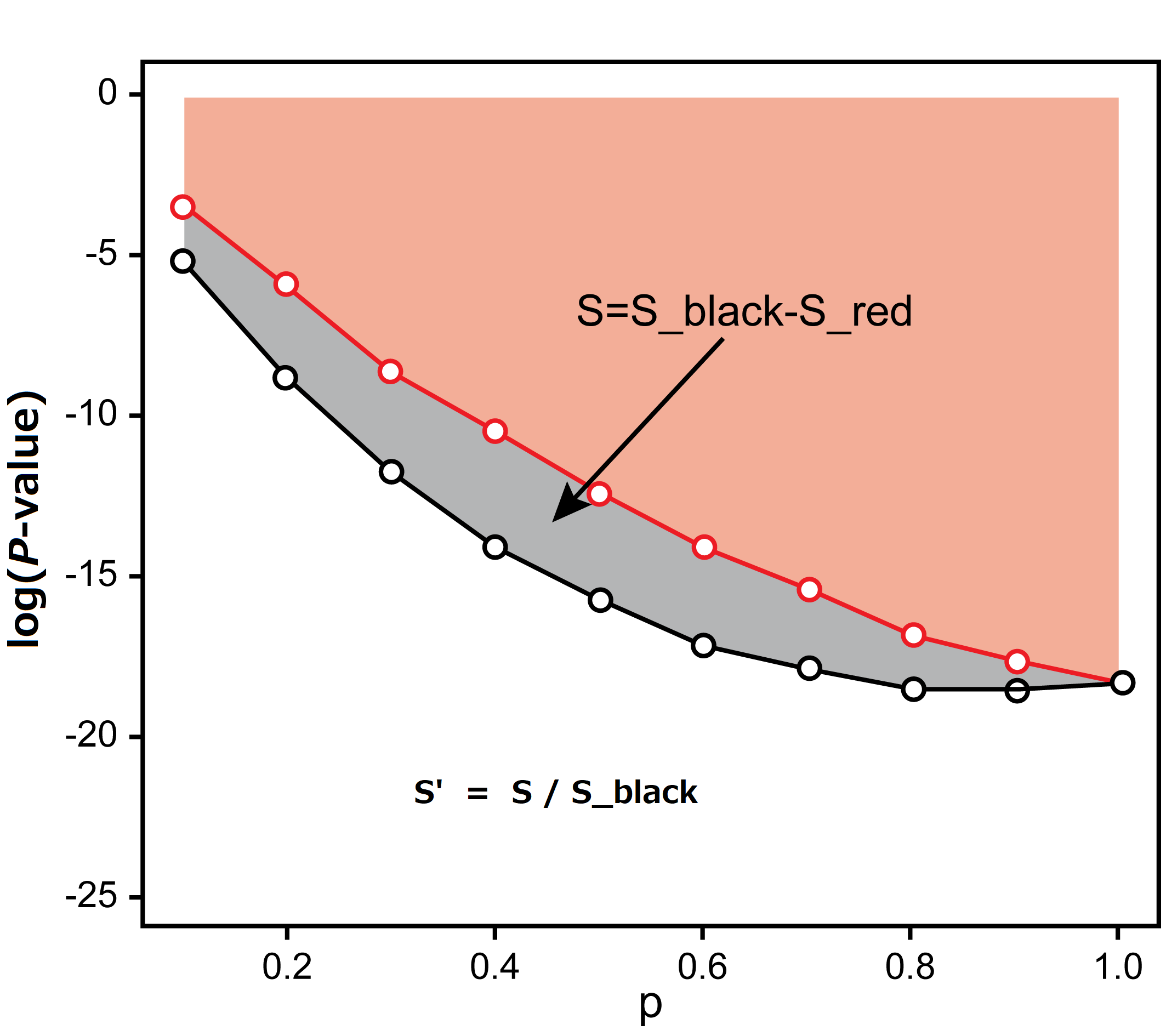

Supplement: Supplementary file 1 — Calculating the relative difference (S′) in (p, log(P-value)) plots. The y-axis represents the log(P-value), and the x-axis represents the risk allele frequency (p). The relative difference (S′) was defined as the ratio of the area between the black line (minor allele as the risk allele) and the red line (major allele as the risk allele) to the maximum area surrounded by the black and red lines. (TIFF 479 kb) [file 12920_2018_322_MOESM1_ESM.tif]

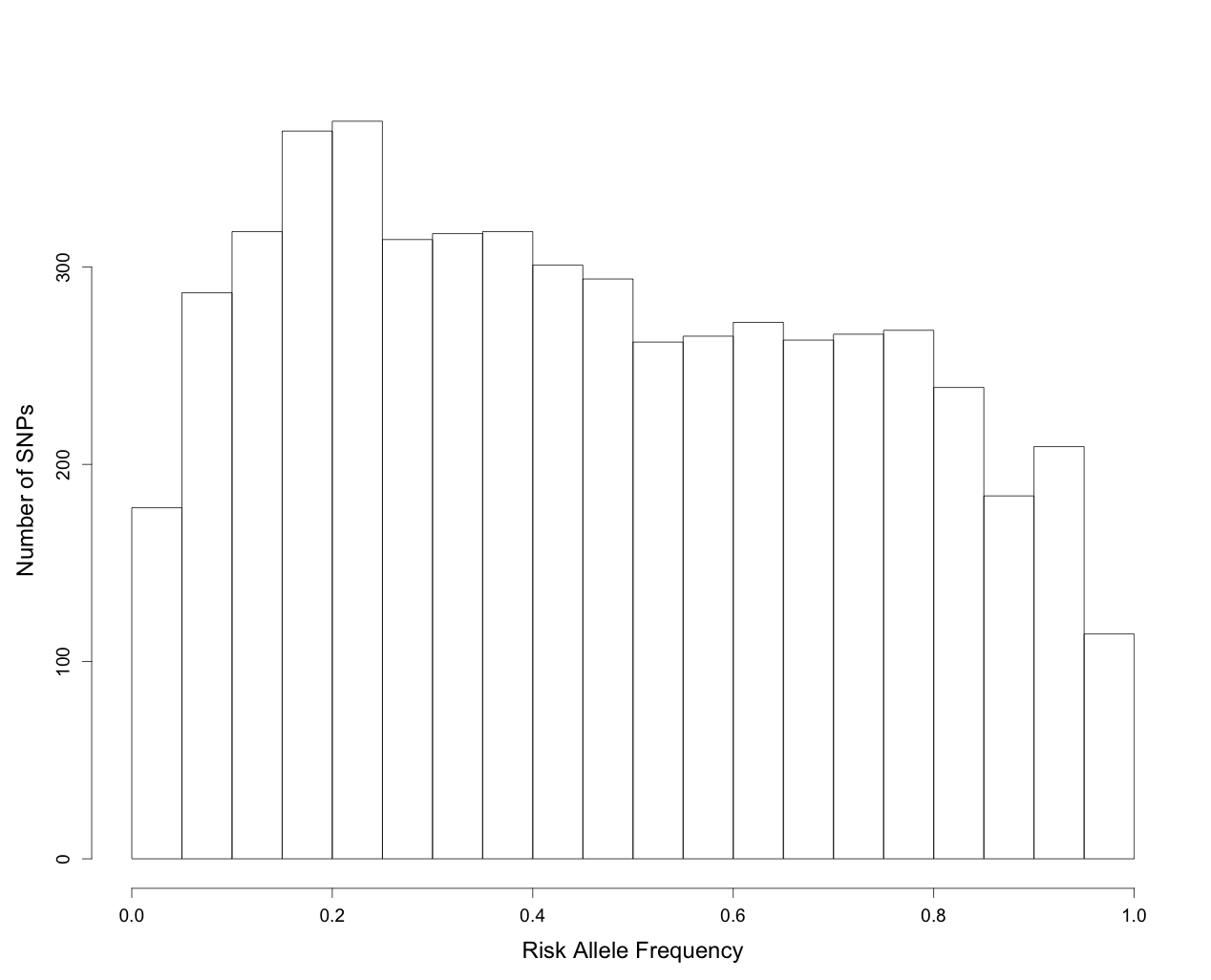

Supplement: Supplementary file 3 — Risk allele frequencies of LD block SNVs for 213 diseases extracted from the VARIMED database of European association studies. (TIFF 156 kb) [file 12920_2018_322_MOESM3_ESM.tif]

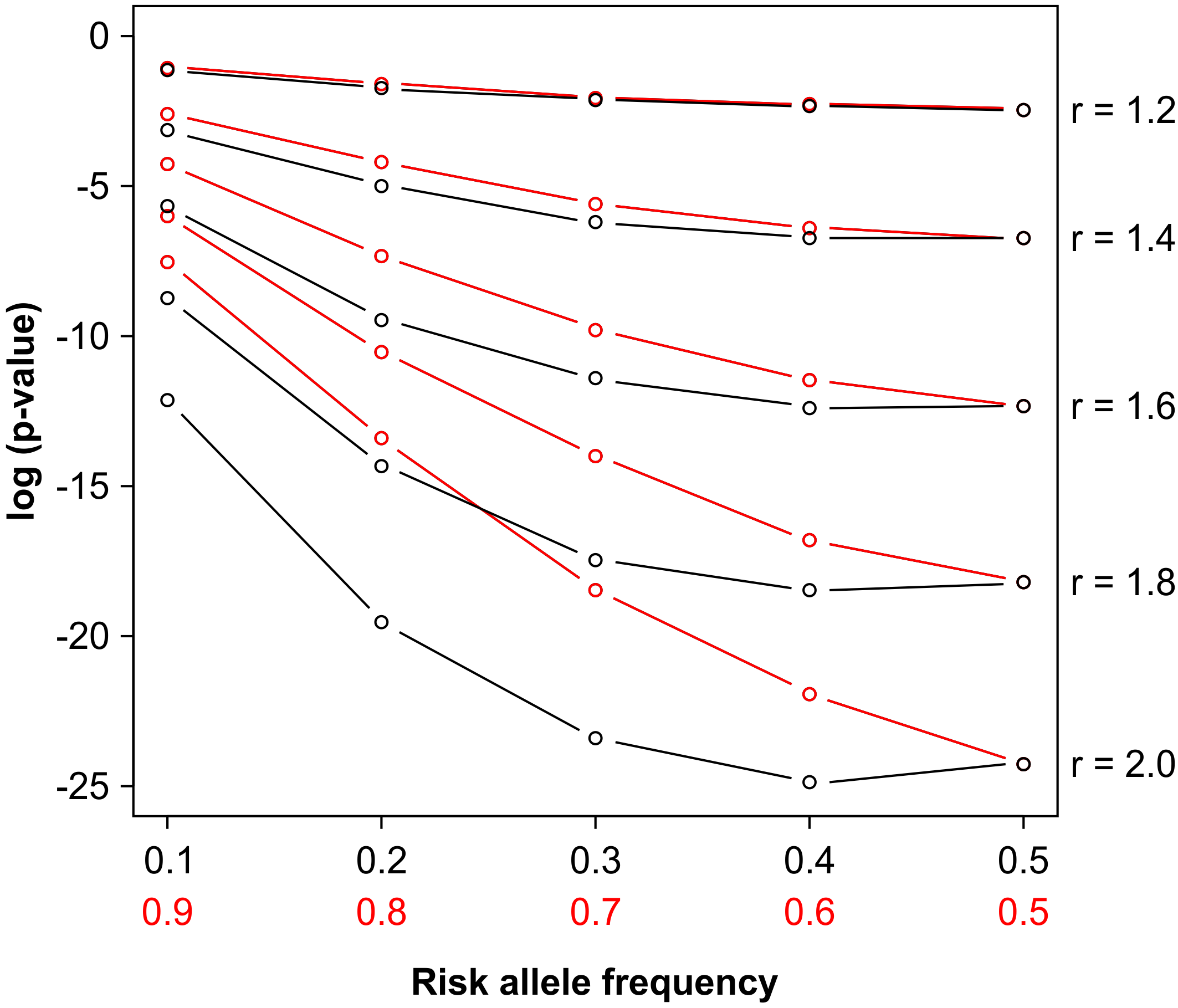

Supplement: Supplementary file 4 — Log-transformed P-values of association tests determined by logistic regression analysis with penetrance for genotype with the lowest risk of 0.01 and odds ratio increments of 0.2. The sample size was 1000 for both cases and controls. The penetrance for the genotype with the lowest risk was 0.01, and the odds ratio (r) ranged from 1.1 to 2.0 in increments of 0.2. Red and black circles/lines indicate values for risk allele frequencies shown in red and black, respectively. (TIFF 570 kb) [file 12920_2018_322_MOESM4_ESM.tif]

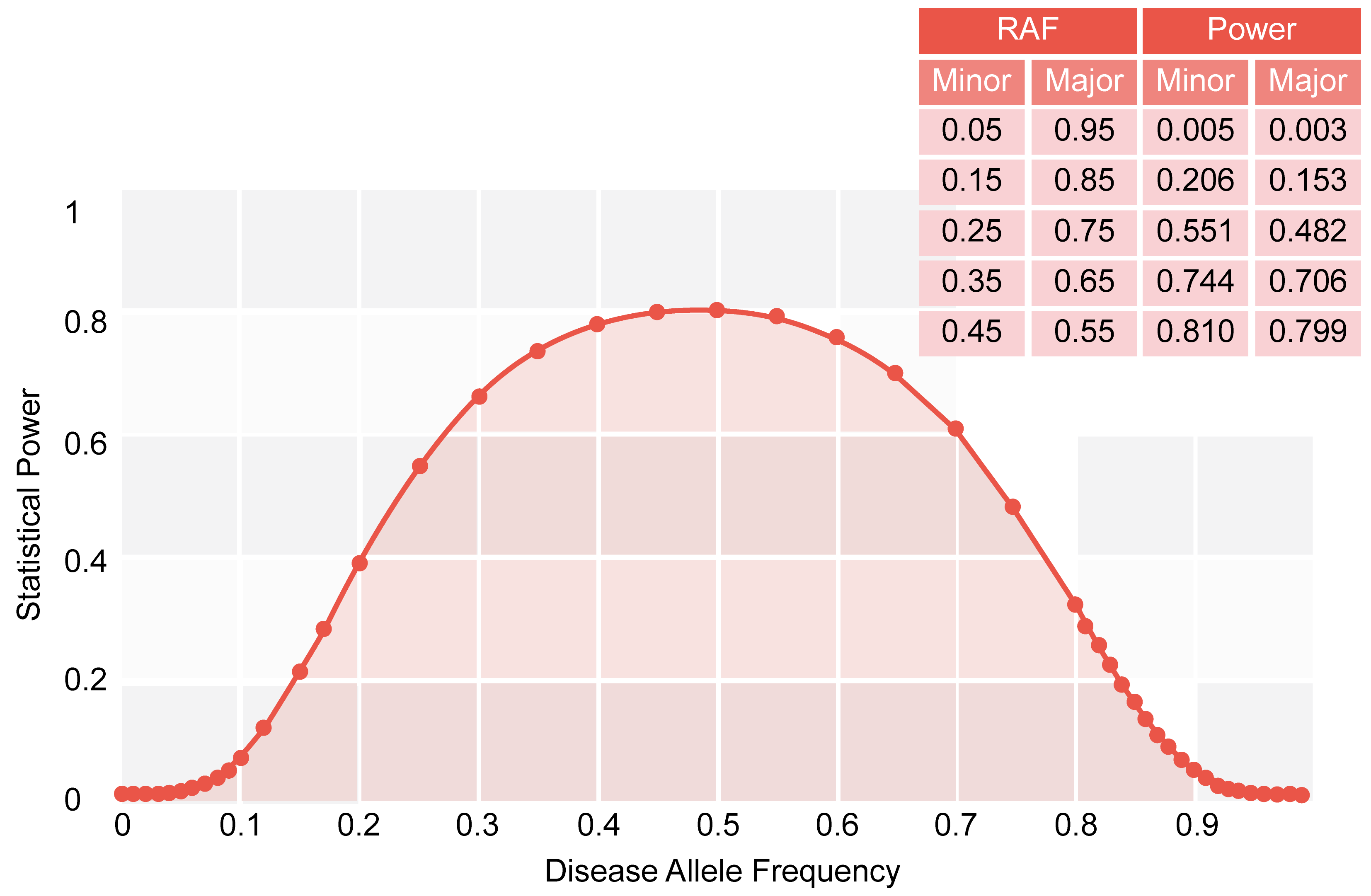

Supplement: Supplementary file 5 — Statistical power analysis for type 1 diabetes study [17]. The number of cases was 7514, and the number of controls was 9045. We assumed that the significance level of the study design was 5.0 × 10− 8, the prevalence was 0.002, the genotype relative risk was 1.15, and the disease model was multiplicative. The graph shows the relationships between disease allele frequency (x-axis) and the statistical power (y-axis). The right top table shows the difference between the statistical power in the minor and major risk allele frequency (p = 0.05 vs. p = 0.95, p = 0.15 vs. p = 0.85, p = 0.25 vs. p = 0.75, p = 0.35 vs. p = 0.65, and p = 0.45 vs. p = 0.55). (TIFF 689 kb) [file 12920_2018_322_MOESM5_ESM.tif]

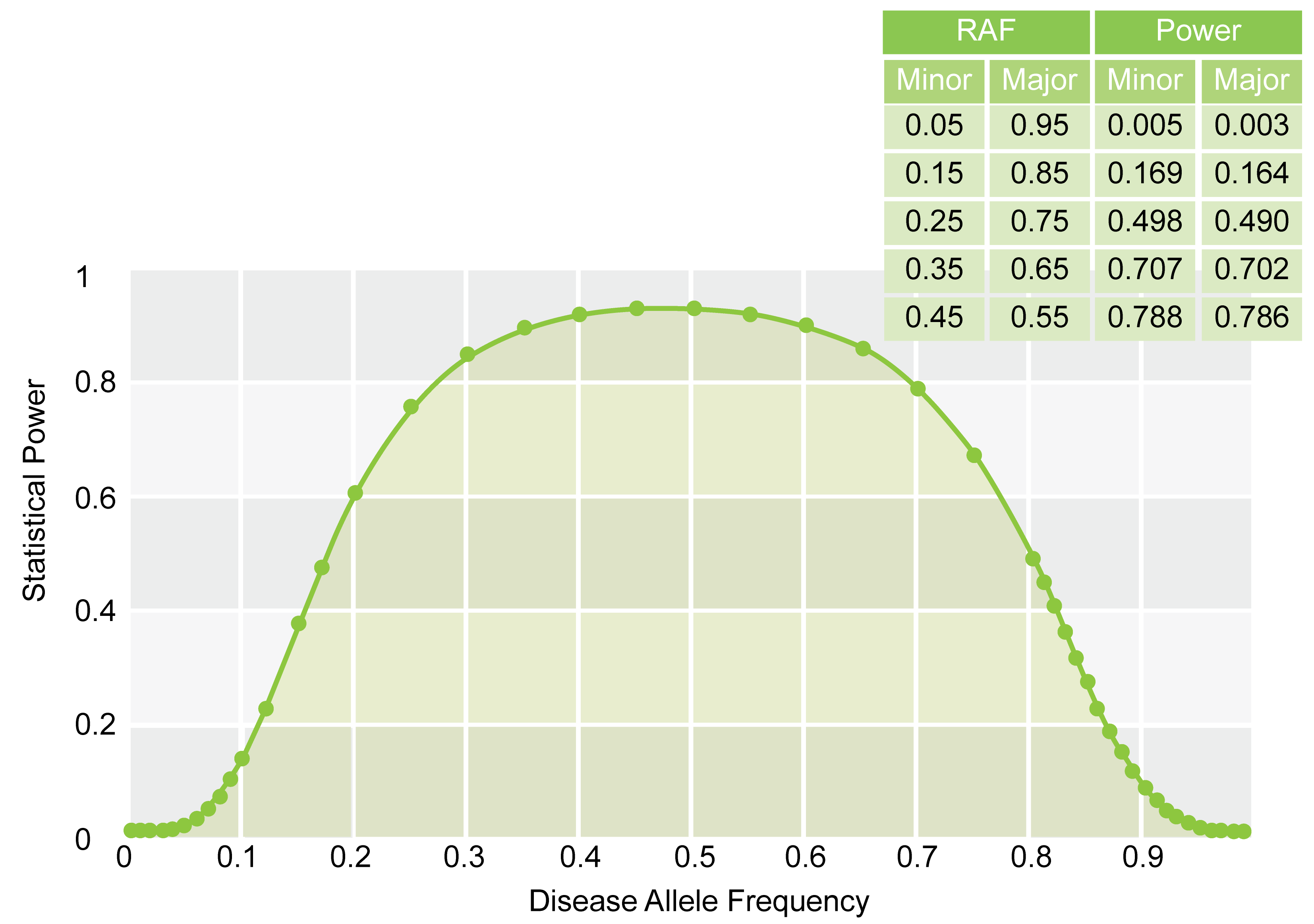

Supplement: Supplementary file 6 — Statistical power analysis for type 2 diabetes study [20]. The number of cases was 4595, and the number of controls was 5579. We assumed that the significance level of the study design was 5.0 × 10− 8, the prevalence was 0.073, the genotype relative risk was 1.2, and the disease model was multiplicative. The graph shows the relationships between disease allele frequency (x-axis) and the statistical power (y-axis). The right top table shows the difference between the statistical power in the minor and the major risk allele frequency (p = 0.05 vs. p = 0.95, p = 0.15 vs. p = 0.85, p = 0.25 vs. p = 0.75, p = 0.35 vs. p = 0.65, and p = 0.45 vs. p = 0.55). (TIFF 757 kb) [file 12920_2018_322_MOESM6_ESM.tif]

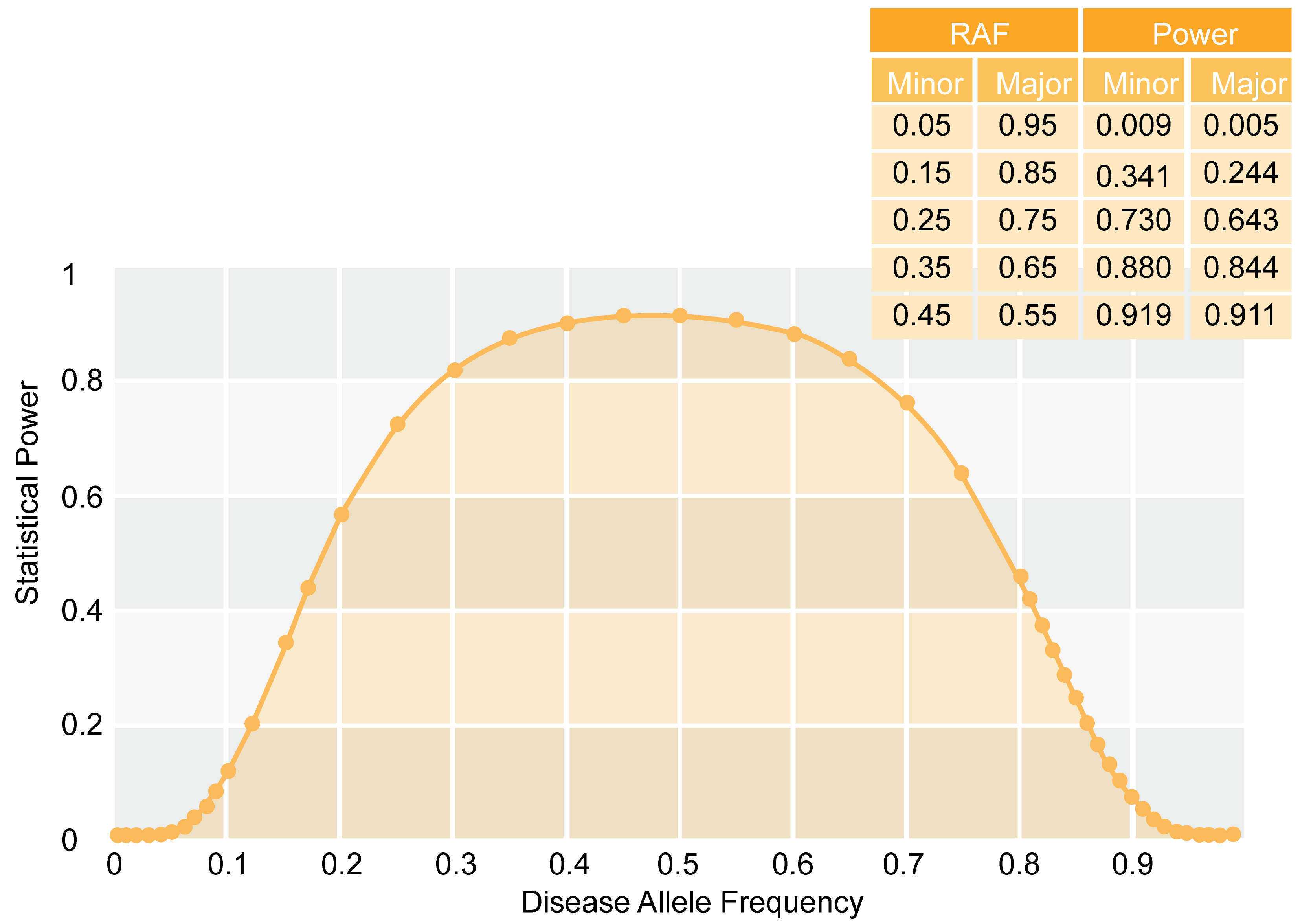

Supplement: Supplementary file 7 — Statistical power analysis for schizophrenia study [21]. The number of cases was 5001, and the number of controls was 6243. We assumed that the significance level of the study design was 5.0 × 10− 8, the prevalence was 0.01, the genotype relative risk was 1.2, and the disease model was multiplicative. The graph shows the relationships between disease allele frequency (x-axis) and the statistical power (y-axis). The right top table shows the difference between the statistical power in the minor and major risk allele frequency (p = 0.05 vs. p = 0.95, p = 0.15 vs. p = 0.85, p = 0.25 vs. p = 0.75, p = 0.35 vs. p = 0.65, and p = 0.45 vs. p = 0.55). (TIFF 777 kb) [file 12920_2018_322_MOESM7_ESM.tif]

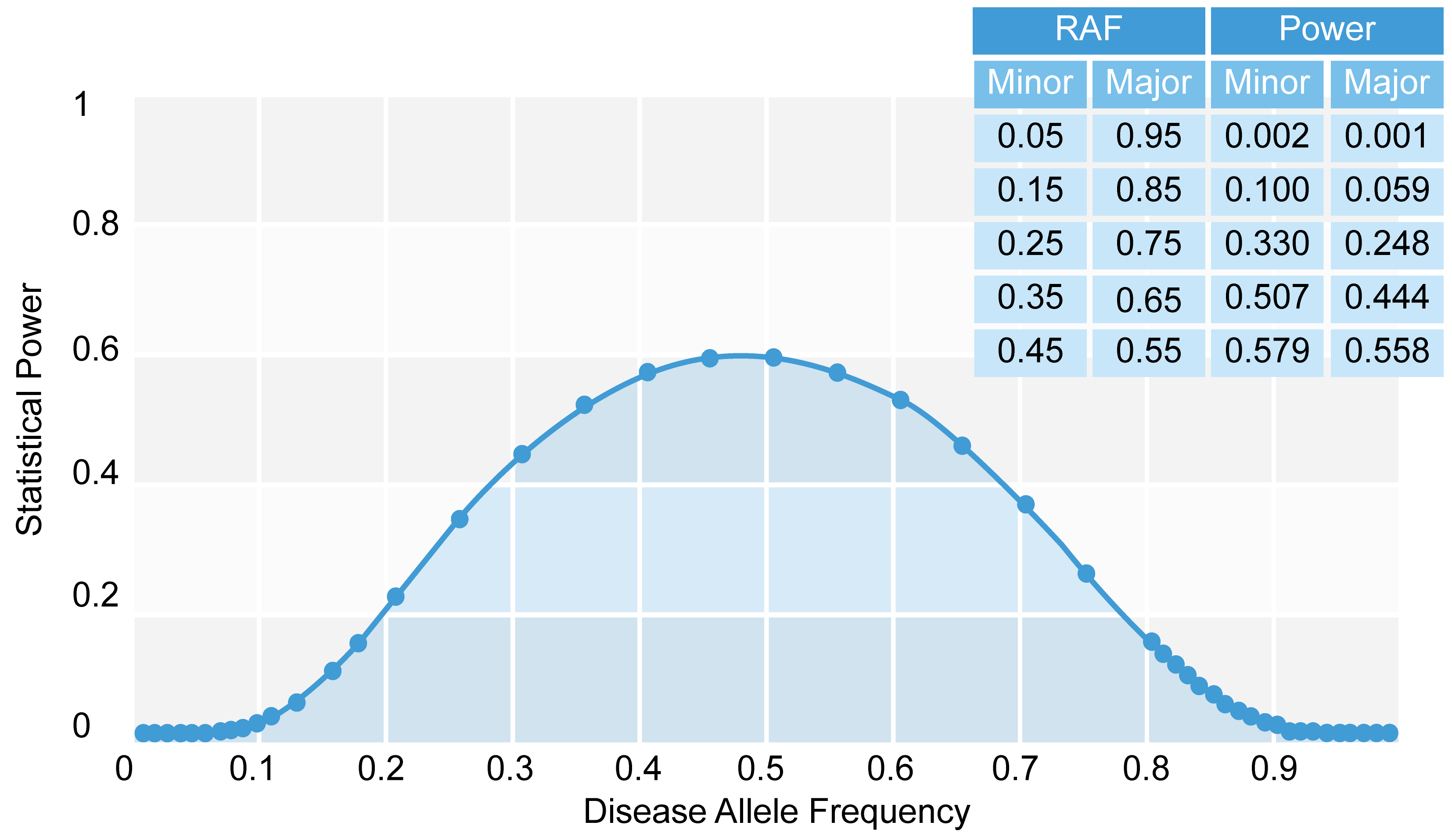

Supplement: Supplementary file 8 — Statistical power analysis for myopia study [18]. The number of cases was 190, and the number of controls was 1064. We assumed that the significance level of the study design was 5.0 × 10− 8, the prevalence was 0.25, the genotype relative risk was 1.6, and the disease model was multiplicative. The graph shows the relationships between disease allele frequency (x-axis) and the statistical power (y-axis). The right top table shows the difference between the statistical power in the minor and major risk allele frequency (p = 0.05 vs. p = 0.95, p = 0.15 vs. p = 0.85, p = 0.25 vs. p = 0.75, p = 0.35 vs. p = 0.65, and p = 0.45 vs. p = 0.55). (TIFF 646 kb) [file 12920_2018_322_MOESM8_ESM.tif]
