# Supplementary material for: Are minor alleles more likely to be risk alleles?
Source: BMC Med Genomics. 2018 Jan 19;11:3. doi: 10.1186/s12920-018-0322-5 (PMC5775585; doi:10.1186/s12920-018-0322-5)
Supplement: Supplementary file 9 — Comparisons of observed and expected proportions of SNVs whose risk alleles are minor alleles in sudden cardiac arrest. Table S2. Comparisons of observed and expected proportions of SNVs whose risk alleles are minor alleles in systemic lupus erythematosus. (DOCX 16 kb) [file 12920_2018_322_MOESM9_ESM.docx]

**Additional file 9**

**Table S1 Comparisons of observed and expected proportions of SNVs whose risk alleles are minor alleles in sudden cardiac arrest.**

| MAF interval | SNVs whose risk alleles are minor alleles | SNVs whose risk alleles are major allele | Observed proportion^a^ | Expected proportion^b^ | *P*-value^c^ (Considering the GWAS's power imbalance) | *Lower limit^d^ (Considering the GWAS's power imbalance)* | *Upper limit^d^ (Considering the GWAS's power imbalance)* |
| --- | --- | --- | --- | --- | --- | --- | --- |
|  |  |  |  |  |  |  |  |
| (0−0.1) | 4 | 0 | 1 | 0.53 | 0.12 | 0.39 | 1.00 |
| (0.1−0.2) | 8 | 0 | 1 | 0.50 | 0.0078*^e^ | 0.63 | 1.00 |
| (0.2−0.3) | 1 | 0 | 1 | 0.50 | 1.00 | 0.03 | 1.00 |
| (0.3−0.4) | 0 | 0 | NA | 0.50 | NA | NA | NA |
| (0.4−0.5) | 0 | 0 | NA | 0.50 | NA | NA | NA |

The parameters for the statistical power calculation were set according to　the following studies.

(1) 12 SNVs, Cases = 100, controls = 520, significance level = 5.0E-08, prevalence = 0.15, genotype relative risk = 1.60 [1].

(2) 1 SNV, cases = 1283, controls = 2000, significance level = 5.0E-08, prevalence = 0.15, genotype relative risk = 1.50 [2].

The statistical powers of detecting minor alleles and major alleles in MAF intervals (0−0.1), (0.1−0.2), (0.3−0.4), and (0.4−0.5) were calculated for each study.

Expected proportions for the MAF intervals were calculated based on the statistical power calculation for each study.

Average expected proportions of the 2 studies are reported in the table.

^a^Proportion of SNVs whose risk alleles are minor alleles.

^b^Expected proportion of SNVs whose risk alleles are minor alleles (average of 2 studies).

^c^*P*-value for the binomial test with the null hypothesis that the observed proportion is the expected proportion.

^d^Lower and upper limits of the 95% confidence interval for the proportion by the Clopper–Pearson method with the null hypothesis that the observed proportion is the expected proportion.

^e^**P*-value ≤ 0.01

1. Aouizerat BE, Vittinghoff E, Musone SL, Pawlikowska L, Kwok PY, Olgin JE, et al. GWAS for discovery and replication of genetic loci associated with sudden cardiac arrest in patients with coronary artery disease. BMC Cardiovasc Disord. 2011;11:29.

2. Arking DE, Junttila MJ, Goyette P, Huertas-Vazquez A, Eijgelsheim M, Blom MT, et al. Identification of a sudden cardiac death susceptibility locus at 2q24.2 through genome-wide association in European ancestry individuals. PLoS Genet. 2011;7:e1002158.

Table S2 Comparisons of observed and expected proportions of SNVs whose risk alleles are minor alleles in systemic lupus erythematosus.

| MAF interval | SNVs whose risk alleles are minor alleles | SNVs whose risk alleles are major alleles | Observed proportion^a^ | Expected proportion^b^ | *P*-value^c^ (Considering the GWAS’s power imbalance) | *Lower limit^d^ (Considering the GWAS’s power imbalance)* | *Upper limit^d^ (Considering the GWAS’s power imbalance)* |
| --- | --- | --- | --- | --- | --- | --- | --- |
|  |  |  |  |  |  |  |  |
| (0, 0.1) | 7 | 0 | 1 | 0.68 | 0.10 | 0.59 | 1.00 |
| (0.1, 0.2) | 13 | 0 | 1 | 0.53 | 0.00031* | 0.75 | 1.00 |
| (0.2, 0.3) | 5 | 0 | 1 | 0.53 | 0.062 | 0.47 | 1.00 |
| (0.3, 0.4) | 5 | 0 | 1 | 0.51 | 0.062 | 0.47 | 1.00 |
| (0.4, 0.5) | 1 | 0 | 1 | 0.5 | 1 | 0.025 | 1.00 |

The parameters for the statistical power calculation were according to the following studies.

(1) 5 SNVs, Cases = 1,311, controls = 3,340, significance level = 5.0E-08, prevalence = 0.001, genotype relative risk = 1.3 [1].

(2) 1 SNV, cases = 279, controls = 515, significance level = 5.0E-08, prevalence = 0.001, genotype relative risk = 2.3 [2].

(3) 3 SNVs, cases = 431, controls = 2155, significance level = 5.0E-08, prevalence = 0.001, genotype relative risk = 1.5 [3].

(4) 5 SNVs, cases = 720, controls = 2,337, significance level = 5.0E-08, prevalence = 0.001, genotype relative risk = 1.5 [4].

(5) 11 SNVs, cases = 725, controls = 2,438, significance level = 5.0E-08, prevalence = 0.001, genotype relative risk = 1.9 [5].

(6) 7 SNVs, cases = 811, controls = 906, significance level = 5.0E-08, prevalence = 0.001, genotype relative risk = 1.4 [6].

The statistical powers of detecting minor alleles and major alleles in MAF intervals (0−0.1), (0.1−0.2), (0.3−0.4), and (0.4−0.5) were calculated for each study. Expected proportions for the MAF intervals were calculated based on the statistical power calculation for each study. Average expected proportions of the 6 studies are reported in the table.

^a^Proportion of SNVs whose risk alleles are minor alleles.

^b^Expected proportion of SNVs whose risk alleles are minor alleles (average of 6 studies).

^c^*P*-value for the binomial test with the null hypothesis that the observed proportion is the expected proportion.

^d^Lower and upper limits of the 95% confidence interval for the proportion by the Clopper–Pearson method with the null hypothesis that the observed proportion is the expected proportion.

^e^**P*-value ≤ 0.01

1. Hom G, Graham RR, Modrek B, Taylor KE, Ortmann W, Garnier S, et al. Association of systemic lupus erythematosus with C8orf13-BLK and ITGAM-ITGAX. N Engl J Med. 2008;358:900-9.

2. Kozyrev SV, Abelson AK, Wojcik J, Zaghlool A, Linga Reddy MV, Sanchez E, et al. Functional variants in the B-cell gene BANK1 are associated with systemic lupus erythematosus. Nat Genet. 2008;40:211-6.

3. Graham RR, Cotsapas C, Davies L, Hackett R, Lessard CJ, Leon JM, et al. Genetic variants near TNFAIP3 on 6q23 are associated with systemic lupus erythematosus. Nat Genet. 2008;40:1059- 61.

4. International Consortium for Systemic Lupus Erythematosus Genetics (SLEGEN), Harley JB, Alarcón- Riquelme ME, Criswell LA, Jacob CO, Kimberly RP, et al. Genome-wide association scan in women with systemic lupus erythematosus identifies susceptibility variants in ITGAM, PXK, KIAA1542 and other loci. Nat Genet. 2008;40:204-10.

5. Armstrong DL, Zidovetzki R, Alarcón-Riquelme ME, Tsao BP, Criswell LA, Kimberly RP, et al. GWAS identifies novel SLE susceptibility genes and explains the association of the HLA region. Genes Immun. 2014;15:347-54.

6. Chung SA, Taylor KE, Graham RR, Nititham J, Lee AT, Ortmann WA, et al. Differential genetic associations for systemic lupus erythematosus based on anti-dsDNA autoantibody production. PLoS Genet. 2011;7:e1001323.
